# Cardiac adaptations in early equine pregnancy: heart rate elevation without heart rate variability alteration in Thai native crossbred mares

**DOI:** 10.14202/vetworld.2025.2590-2597

**Published:** 2025-09-06

**Authors:** Sutheema Suwannarueang, Wanpitak Pongkan, Theerapong Pontaema, Wootichai Kenchaiwong, Pongphol Pongthaisong, Chayanon Chompoosan, Wichaporn Lerdweeraphon

**Affiliations:** 1 Applied Animal Physiology Research Unit, Faculty of Veterinary Sciences, Mahasarakham University, Mahasarakham, 44000, Thailand; 2 Department of Veterinary Medicine, Faculty of Veterinary Medicine, Chiang Mai University, Chiang Mai, 50100, Thailand

**Keywords:** heart rate, heart rate variability, pregnancy, mares, vasovagal tonus index, autonomic regulation

## Abstract

**Background and Aim:**

Pregnancy induces significant anatomical and physiological changes, many of which are regulated by the autonomic nervous system (ANS). Heart rate variability (HRV) is a well-established non-invasive tool for assessing ANS activity. While changes in heart rate (HR) and HRV during the third-trimester of equine pregnancy are documented, there is limited understanding of cardiac autonomic adaptations during the early stages of gestation. This study aimed to compare HR and time-domain HRV parameters between healthy non-pregnant mares and those in the first and second trimesters of pregnancy.

**Materials and Methods:**

A total of 45 Thai native crossbred mares were enrolled and divided into three groups: Non-pregnant (n = 5), first-trimester pregnant (0–114 days; n = 18), and second-trimester pregnant (115–226 days; n = 22). All mares were clinically healthy and free from cardiac abnormalities. Electrocardiographic data were collected using a Holter electrocardiogram system over a 15 min period at rest, and HRV was analyzed using time-domain measures: Standard deviation of all NN intervals (SDNN), SDNN index, root mean square of successive differences, standard deviation of 5-min mean NN intervals, percentage of successive NN intervals >50 ms, and vasovagal tonus index (VVTI). Data were analyzed using Kruskal–Wallis and Mann–Whitney *U*-tests.

**Results:**

HR was significantly higher in first-trimester pregnant mares compared to non-pregnant mares (p < 0.05), and even higher in the second-trimester compared to the first (p < 0.05). However, there were no significant differences among the groups in any of the HRV parameters or VVTI.

**Conclusion:**

The findings indicate that cardiovascular adaptation during early pregnancy in mares is characterized by a progressive increase in HR, likely reflecting increased cardiac output to support fetal development. However, the lack of significant changes in time-domain HRV parameters and VVTI suggests that ANS balance is maintained during the first and second trimesters. These results provide valuable reference values for equine reproductive monitoring and contribute to a better understanding of physiological changes in early gestation.

## INTRODUCTION

Pregnancy triggers a cascade of anatomical and physiological changes in the mare to support the developing fetus [1]. These changes begin shortly after conception and affect multiple organ systems. The autonomic nervous system (ANS) plays a pivotal role in orchestrating these adaptations, particularly through modulation of cardiac output (CO) and vascular resistance [2]. During early pregnancy, CO can increase by up to 20%, with noticeable changes occurring as early as 8 weeks of gestation [3]. Both heart rate (HR) and HR variability (HRV) are significantly altered during pregnancy [4], reflecting the influence of ANS adjustments. HRV serves as a non-invasive marker for evaluating the balance between sympathetic and parasympathetic nervous system activity [5].

In humans, the first-trimester is typically associated with an increase in HR and a reduction in HRV, indicating a shift toward greater sympathetic dominance and reduced parasympathetic activity [6]. However, comparable data on ANS function during early gestation in mares are limited. Understanding the trajectory of normal physiological changes in early pregnancy is essential for distinguishing healthy adaptation from pathological alterations in equine reproduction.

In horses, HRV is a widely accepted, non-invasive approach for assessing physiological and psychological states [7–10], as well as detecting arrhythmias [11]. HRV is defined as the variation in the intervals between successive heartbeats (RR or NN intervals), particularly those occurring between R waves. A specific time-domain index, vasovagal tonus index (VVTI), has proven useful in evaluating sympathovagal balance in horses and offers a simplified method for interpreting HRV in field conditions [12].

A recent study by Chompoosan *et al*. [13] has reported a decline in VVTI and an increase in HR in third-trimester pregnant mares compared with non-pregnant counterparts. Additionally, HR tends to rise before foaling, while HRV diminishes during parturition in smaller mare breeds [14]. Despite these observations, the pattern of HR and HRV changes during the early stages of pregnancy in mares remains unclear.

While numerous studies have explored the cardiovascular adaptations in pregnant mares during the late stages of gestation, especially in the third-trimester, there is a notable lack of data regarding these changes during early pregnancy. Previous research has demonstrated increased HR and decreased HRV in the final trimester, suggesting heightened sympathetic activity and reduced parasympathetic tone. However, these findings are limited to advanced pregnancy stages, and there is insufficient understanding of how early gestational changes, particularly during the first and second trimesters, affect autonomic regulation in mares. Furthermore, although the VVTI has been validated as a practical measure of autonomic tone in horses, its application in monitoring early pregnancy remains underexplored. This knowledge gap restricts veterinarians’ ability to distinguish between normal and pathological cardiovascular responses during the early stages of equine gestation, especially in field conditions where invasive diagnostics may not be feasible.

To address this gap, the present study aimed to investigate the effects of early pregnancy on HR and time-domain HRV parameters in Thai native crossbred mares. Specifically, the objective was to compare HR and HRV indices, including standard deviation of all NN intervals (SDNN), SDNN index, root mean square of successive differences (rMSSD), standard deviation of 5-min mean NN intervals (SDANN), percentage of successive NN intervals >50 ms (pNN50), and VVTI – among healthy non-pregnant mares and mares in their first and second trimesters of pregnancy. By evaluating these physiological markers, this study seeks to enhance understanding of ANS adaptations in early equine pregnancy and establish reference values that can support clinical monitoring, reproductive management, and welfare assessment in broodmares.

## MATERIALS AND METHODS

### Ethical approval

This study was approved by the Institutional Animal Ethics Committee of Mahasarakham University, Thailand (Approval number: IACUC-MSU-44/2024).

### Study period and location

The study was conducted from October to November 2024 at the Husbandry Section of the 2^nd^ Livestock and Agriculture Division, Veterinary and Remount Department, located in Tha Phra Subdistrict, Mueang District, Khon Kaen Province, Thailand.

### Animals and grouping

A total of 45 healthy Thai native crossbred mares were included, with an average age of 7.6 ± 2.5 years and body weight of 382.4 ± 71.0 kg. Pregnancy status and gestational age were determined using breeding history and transrectal ultrasonography. Based on gestational age, animals were allocated into three groups:

- Non-pregnant mares (n = 5)
- First-trimester pregnant mares (0–114 days; n = 18)
- Second-trimester pregnant mares (115–226 days; n = 22) [15].

All mares were free from cardiac abnormalities (e.g., murmurs, arrhythmias) and showed no pregnancy-related complications. The mares were housed under identical management conditions at a single facility (16°20’18.6”N, 102°48’23.5”E) and were fed *Brachiaria mutica* (para grass) *ad libitum* along with a 16% protein commercial concentrate (Charoen Pokphand Group, Bangkok, Thailand) at 1.6 kg/100 kg body weight. Water and mineral salts were also provided *ad libitum*. All recordings were performed between 2:00 and 3:00 p.m.

### Experimental design and data collection

This study aimed to evaluate HR and time-domain HRV parameters, including SDNN, SDNN index, rMSSD, SDANN, pNN50, and VVTI. Before data collection, all mares underwent a physical examination and were allowed to stand quietly for 5-min with a halter to minimize excitement.

### Electrocardiographic recording

**Figure 1:**
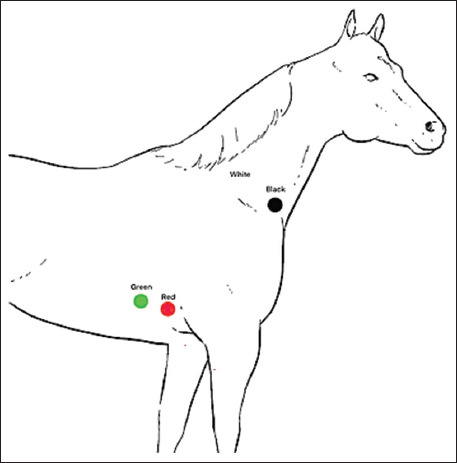
Electrode placement of the modified base-apex lead method [16].

Electrocardiogram (ECG) recordings were acquired using a Holter ECG system (SE-2012, EDAN Instruments Inc., China), which features a 3-channel display and settings of 25 mm/s paper speed and 10 mm/mV sensitivity. Mares were not sedated. Electrodes were attached using a modified base-apex lead system [16] as shown in Figure 1:

- Green and red electrodes: 5^th^ intercostal space (right side)
- White and black electrodes: Right jugular furrow.

Each mare was recorded at rest for 15 min. The ECG data were saved to a secure digital card and transferred to a computer for analysis using Holter software.

### HR and HRV analysis

HR and HRV parameters were automatically calculated from RR intervals using Holter software. Artifacts in the recordings were manually corrected before analysis. The following time-domain HRV metrics were computed:

- SDNN: Standard deviation of all RR intervals
- SDNN index: Mean of 5-min SDNN values
- rMSSD: Root mean square of successive RR differences
- SDANN: Standard deviation of 5-min mean RR intervals
- pNN50: Percentage of successive RR intervals >50 ms
- VVTI: Calculated using the first 20 consecutive RR intervals with the formula:

VVTI = NL [VAR (RR_1_–RR_20_)],

where *NL* is the natural logarithm and *VAR* represents variance in milliseconds [17].

### Statistical analysis

The normality of HRV parameters was assessed using Q–Q plots and the Shapiro–Wilk test. Since most variables were not normally distributed, non-parametric methods (Kruskal–Wallis test) were applied. VVTI values were log-transformed based on the method by Pereira *et al*. [17] and analyzed using a generalized linear model. Significant differences were followed by *post hoc* comparisons using the Mann–Whitney U test. All analyses were performed using SAS University Edition (SAS/STAT, SAS Institute Inc., NC, USA). Data are presented as means, and statistical significance was set at p < 0.05.

## RESULTS

### Normality testing of HRV parameters

HRV parameters were assessed for normality using the Shapiro–Wilk test. The variables SDNN, SDNN index, rMSSD, SDANN, and pNN50 all exhibited high coefficients of variation (CV) (CV > 50%), indicating substantial variability among samples (Table 1). The Shapiro–Wilk test results showed that these parameters were not normally distributed (p < 0.05), necessitating the use of non-parametric statistical methods for further analysis.

**Table 1:** Descriptive statistics of tests for normality of HRV variables in the 45 mares by Shapiro–Wilk test.

Items	HR	SDNN	SDNN_idx	rMSSD	SDANN	pNN50
Descriptive statistics						
n	45	45	45	45	45	45
Mean	47.2	168.5	154.5	87.7	44.6	18.4
Standard deviation	11.0	118.7	116.7	56.6	37.0	12.2
Skewness	1.7	1.4	1.5	1.4	1.8	0.6
Coefficients variation	23.3	70.5	75.5	64.6	82.9	63.9
Standard error mean	1.6	17.7	17.4	8.4	5.5	1.8
Median	45.0	124.0	110.0	76.0	29.5	16.0
Mode	42.0	121.0	38.0	36.0	60.0	13.0
Maximum	88.0	539.0	529.0	250.0	182.0	48.0
Minimum	33.0	35.0	34.0	30.0	4.0	1.0
Tests for normality						
Shapiro-Wilk (Pr < W)	0.85 (<0.0001)*	0.85 (<0.0001)*	0.82 (<0.0001)*	0.82 (<0.0001)*	0.83 (<0.0001)*	0.94 (0.03)*

* The significance of test normality was p of <0.05. HR = Heart rate, rMSSD = Root mean square of successive differences, SDNN = Standard deviation of all NN intervals, SDANN=Standard deviation of 5-min mean NN intervals, pNN50 = Percentage of successive NN intervals >50 ms

### Comparison of HR among groups

HR values significantly differed among the groups. The mean HR was significantly higher in second-trimester pregnant mares (51 ± 12.68 bpm) compared to first-trimester mares (46 ± 8.79 bpm) (p < 0.05). Additionally, first-trimester pregnant mares had a significantly higher HR than non-pregnant mares (39 ± 5.87 bpm) (p < 0.05) (Table 2). These findings suggest a progressive increase in maternal HR with advancing gestation.

**Table 2:** Comparison of heart rate variability variables (time-domain analysis) between pregnant (first and second trimesters) and non-pregnant mares.

HRV variables	Trimesters of pregnancy	n	Wilcoxon scores		Kruskal–Wallis test	
	
			Sum of the scores	Mean score	Chi-square (df = 2)	Pr > Chi-square
HR	First (0–114 d)	18	385.0	21.4^ac^	6.84	0.032*
	Second (115–226 d)	22	596.5	27.1^ab^		
	Non-pregnant	5	53.5	10.7^c^		
SDNN	First (0–114 d)	18	366.0	20.3	3.25	0.20
	Second (115–226 d)	22	507.5	23.1		
	Non-pregnant	5	161.5	32.3		
SDNN_idx	First (0–114 d)	18	363.0	20.2	3.15	0.21
	Second (115–226 d)	22	512.5	23.3		
	Non-pregnant	5	159.5	31.9		
rMSSD	First (0–114 d)	18	366.0	20.3	1.93	0.38
	Second (115–226 d)	22	523.0	23.8		
	Non-pregnant	5	146.0	29.2		
SDANN	First (0–114 d)	18	375.0	20.8	1.13	0.57
	Second (115–226 d)	22	552.5	25.1		
	Non-pregnant	5	107.5	21.5		
pNN50	First (0–114 d)	18	462.5	25.7	1.34	0.51
	Second (115–226 d)	22	459.5	20.9		
	Non-pregnant	5	113.0	22.6		

* The letter difference was tested by Mann–Whitney U test with a p < 0.05 was considered statistically significant. HR = Heart rate, rMSSD = Root mean square of successive differences, SDNN = Standard deviation of all NN intervals, SDANN = Standard deviation of 5-min mean NN intervals, pNN50 = Percentage of successive NN intervals >50 ms

### Comparison of time-domain HRV parameters and VVTI

**Figure 2:**
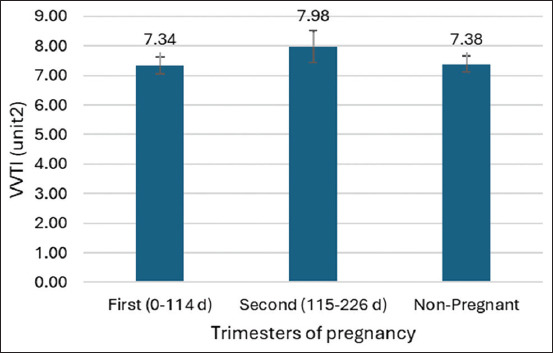
Comparison of the ls-means vasovagal tonus index parameters in each trimester of the pregnancy of mares using a generalized linear model.

Despite the significant increase in HR, no significant differences were observed in time-domain HRV parameters – SDNN, SDNN index, rMSSD, SDANN, and pNN50 – across the three groups. Similarly, VVTI values did not significantly differ among non-pregnant, first-trimester, and second-trimester mares (Tables 2 and 3; Figure 2). These results suggest that while HR increases during early pregnancy, the autonomic balance, as reflected by HRV and VVTI, remains stable.

**Table 3:** Comparison of the vasovagal tonus index in non-pregnant and pregnant mares.

Trimesters of pregnancy	n	Wilcoxon scores		Kruskal-Wallis test	
	
		Sum of the scores	Mean score	Chi-square (df = 2)	Pr > Chi-square
First (0–114 d)	18	397	22.05		
Second (115–226 d)	22	451	21.47		
Non-pregnant	5	142	28.40	1.21	0.54

A p < 0.05 was considered statistically significant

## DISCUSSION

### Comparison with previous findings

A previous study by Chompoosan *et al*. [13] has reported an increase in HR and a reduction in VVTI in mares during the third-trimester of pregnancy (average 235 ± 35.8 days) compared to non-pregnant mares, suggesting heightened sympathetic activity in late gestation. However, evidence regarding HR and HRV changes during early pregnancy remains scarce. This study hypothesized that HR would increase and HRV would decrease in mares during the first and second trimesters (average 121.5 ± 47.9 days), compared to healthy non-pregnant mares. The primary finding confirmed an increase in HR during early pregnancy, but no significant changes were observed in time-domain HRV parameters.

### HR elevation and physiological adaptation

HR was significantly higher in first-trimester mares than in non-pregnant mares and was highest in second-trimester mares. This pattern may be attributed to ongoing fetal development and the associated physiological demands during early gestation [18]. Cardiovascular changes are known to begin early in pregnancy, with CO increasing by approximately 20% by the 8^th^ week of gestation [3]. This increase is likely facilitated by a shift toward sympathetic dominance and reduced parasympathetic tone, allowing for enhanced uteroplacental perfusion [3, 19, 20]. In the current study involving medium-sized Thai native crossbred mares, second-trimester HR (50.4 ± 13.3 bpm) was higher than first-trimester HR (45.7 ± 8.8 bpm), aligning with findings in both horses [14, 21] and humans [4].

### Stability of HRV parameters during early pregnancy

Although a clear increase in HR was observed, time-domain HRV parameters (SDNN, SDNN index, rMSSD, SDANN, and pNN50) did not differ significantly between pregnant and non-pregnant mares. This finding is consistent with previous studies by Nagel *et al*. [22] and Nagel *et al*. [23] reporting minimal HRV change during gestation in mares. While recent data have shown significantly reduced VVTI in third-trimester pregnant mares [13], our evaluation of VVTI during early pregnancy revealed no significant differences across groups. These results contrast with human studies, where reductions in HRV during both the first [24] and second trimesters [4] have been well documented.

### Species-specific autonomic responses

The discrepancy between species may be explained by fundamental physiological differences. Hemodynamic and autonomic regulatory changes occur during early pregnancy, with increased sympathetic and decreased parasympathetic activity supporting fetal growth [25]. Horses, however, are known to have higher baseline vagal tone than many other species, including dogs, rabbits, mice, and humans, potentially contributing to the stability of HRV despite rising HR [26].

In equine physiology, HRV reductions are more commonly associated with stress [27], physical conditioning [28], or cardiovascular pathology [29], rather than normal pregnancy. The reduction in VVTI observed in third-trimester mares may be due to elevated fetal cortisol and sympathetic activation, reflecting prepartum stress responses [30, 31].

### Implications and future directions

Given that VVTI is a simple yet effective time-domain measure of HRV, its use in third-trimester pregnancy monitoring appears promising. However, during early gestation, it may not be sensitive enough to detect subtle changes in autonomic tone. Hormonal markers – including progesterone, androgens, estrogens, and their metabolites – could complement HR and HRV assessments to better estimate gestational stages, especially when palpation or ultrasonography is not feasible [32].

Hematological and biochemical indicators such as albumin, blood urea nitrogen, and potassium also shift throughout pregnancy and may reflect metabolic adaptations across trimesters [33]. Future research should investigate the combined use of endocrine profiles, hematology, and advanced HRV analyses to refine monitoring strategies for pregnant mares.

### Strengths, limitations, and methodological considerations

Although this study only employed time-domain HRV parameters, newer techniques such as frequency-domain and non-linear HRV analysis are gaining importance in equine research and will be considered in future studies by Physick-Sheard *et al*. [34] and Sanigavatee *et al*. [35]. The use of time-domain methods offered practical advantages for field-based ECG acquisition, particularly given the limited duration of recordings and the small number of non-pregnant control animals. The constrained sample size was due to the primary mission of the participating facility, to breed and maintain pregnant mares, which reduced access to non-pregnant subjects.

## CONCLUSION

This study demonstrated that HR progressively increases during early pregnancy in Thai native crossbred mares, with significantly higher HR observed in first-trimester mares compared to non-pregnant mares and further elevation in the second-trimester. However, time-domain HRV parameters, including SDNN, SDNN index, rMSSD, SDANN, pNN50, and VVTI remained stable across all groups, suggesting that ANS balance is maintained during the early gestational period.

These findings provide important baseline data for the physiological cardiovascular adaptation in mares and support the use of HR monitoring as a simple and non-invasive indicator of early pregnancy progression. The use of a portable Holter ECG system in this study also highlights the practical feasibility of field-based cardiac assessments in equine practice.

A key strength of this research lies in its focus on early pregnancy stages, which have been underrepresented in equine cardiovascular studies. The inclusion of VVTI as a simple HRV measure further enhances its clinical applicability in routine reproductive monitoring.

However, the study is not without limitations. The relatively small number of non-pregnant mares, due to the breeding-focused nature of the study site, may limit broader generalization. Additionally, the analysis was confined to time-domain HRV parameters, without incorporating frequency-domain or non-linear measures, which could provide deeper insight into ANS dynamics.

Future research should consider integrating hormonal, hematological, and biochemical profiles alongside HR and HRV metrics to better predict gestational stages and parturition. Expanded use of advanced HRV analysis techniques and longitudinal monitoring throughout pregnancy would further clarify the trajectory of ANS modulation in mares.

In conclusion, while HR increases early in pregnancy as part of the cardiovascular adaptation, HRV remains unchanged, reflecting a stable autonomic environment in the first and second trimesters. These findings lay the groundwork for improved physiological monitoring and health assessment strategies in pregnant mares under field conditions.

## AUTHORS’ CONTRIBUTIONS

WK: Designed the study and analyzed the data. SS, CC, PP, TP, WP, and WL: Recorded and analyzed the data. WL: Coordinated the study. WL, SS, WP, and WK: Drafted and revised the manuscript. All authors have read and approved the final version of the manuscript.
